# The social genome: Current findings and implications for the study of human genetics

**DOI:** 10.1371/journal.pgen.1006615

**Published:** 2017-03-16

**Authors:** Benjamin W. Domingue, Daniel W. Belsky

**Affiliations:** 1 Graduate School of Education, Stanford University, Stanford California, United States of America; 2 Department of Medicine, Duke University School of Medicine, Durham, North Carolina, United States of America; 3 Duke University Population Research Institute, Duke University, Durham, North Carolina, United States of America; The University of North Carolina at Chapel Hill, UNITED STATES

Human health and diseases are influenced by genes and environments [1]. Recent advances in measures of genetic influences have led to calls for parallel advancements in methods to measure environments [2,3]. The article by Baud et al. [4] in the January 2017 issue suggests one provocative path forward: measure the genomes of proximate individuals.

“Social genetic effects” (SGEs, Baud et al. [4]) arise when an organism’s phenotype is influenced by the genetic makeup of that organism’s social environment. To put it another way, SGEs occur when the genotype of organism A influences the phenotype of organism B [5,6] (Fig 1). Previous SGE research focused on related individuals, especially mothers and offspring [7]. Recent work has explored SGEs in unrelated individuals, with a focus on a narrow range of phenotypes, e.g., sexual display behaviors [8] and body size [9]. The study by Baud et al. [4] suggests SGEs are more pervasive. To the extent results obtained from cage-dwelling mice generalize to free-living humans, findings suggest social genotypes are important environmental parameters.

**Fig 1:**
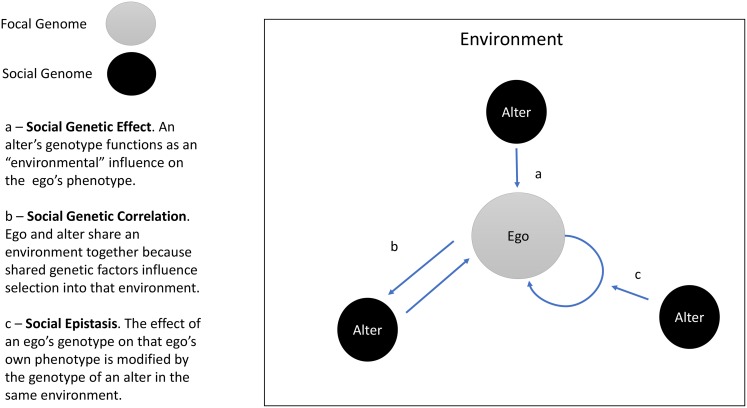
Social Genetic Effects (SGEs), social genetic correlation, and social epistasis. The figure illustrates three social genetic processes: SGEs, social genetic correlation, and social epistasis. The circle labeled “ego” represents the focal individual in an analysis. The circles labeled “alter” represent other individuals within the ego’s social environment. The arrows depict social genetic processes.

## The present study

Baud et al. conducted experiments with cage-dwelling mice to examine the effects of genetic composition of animals’ social environments on psychosocial and physiological phenotypes. Two separate designs evaluated these SGEs. The first design paired inbred C57BL/6J (B6) and DBA/2J (D2) mice as cage-mates under varying social genetic conditions: genetically homogenous (B6/B6 or D2/D2) and genetically heterogeneous (B6/D2). Analysis compared psychosocial and physiological phenotypes between strains and across social genetic conditions. Alongside phenotypic differences between strains, phenotypes also varied depending on social genetic conditions. Differences were primarily found in psychosocial phenotypes—measures of stress and anxiety—but also in wound healing. Pathway analysis of blood gene expression corroborated phenotypic evidence; SGEs on phenotype were reflected in SGEs on patterns of gene expression.

In the second design, the authors examined a large outbred mouse population (*n*> 2,000) in which mice were housed 3–6 to a cage. Using a combination of directly measured genetic data and pedigree information, analysis decomposed variance into direct genetic effects (the effects of a mouse’s own genes) and SGEs. This analysis identified SGEs for more than one third of 117 psychological and physiologic phenotypes at the *p* < 0.05 threshold. SGEs accounted for as much as 29% of phenotypic variance but in general were more modest (5% on average among those traits with *p* < 0.05 SGE).

In a further analysis, the authors attempted to evaluate whether SGEs might confound estimates of direct genetic effects. They report that cage-mates in the outbred-mouse study tended to be more genetically similar as compared to random pairs of mice. Failing to account for this social genetic structure in the data resulted in inflated estimates of heritability for several traits.

In sum, the study by Baud et al. suggests SGEs (i) are pervasive, affecting many phenotypes; (ii) can be pronounced, contributing as much to phenotypic variance in some cases as direct genetic effects; and (iii) are nonignorable, as they may lead to bias in other estimates if not taken into account.

## Implications for research in humans

These experiments with mice highlight opportunities and challenges for social genetic research in humans. One opportunity is to investigate social genotypes as environmental measures. There is already human research investigating social phenotypic effects, e.g., the social “contagion” of obesity (“Are your friends making you fat?”) [10]. But ascertaining causality for social phenotypic influence is challenging [11,12]. Using genetic measures of the social environment to conduct a social version of Mendelian randomization analysis [13] may provide stronger grounds for causal inference.

Baud et al.’s findings further suggest that social genetic factors influencing variation in a given phenotype may be diverse. Thus, genetics previously linked with a particular phenotype may not be the only genetics of interest when considering social genetic effects on that phenotype. Analysis of pleiotropy, e.g., [14,15], may provide a helpful guide in devising more inclusive assays of the social genome.

In addition to direct effects of the social environment/genome, synergies between social and personal genetics are possible. Specifically, social genotyping could be used to study interactions between a person’s genes and the genes of socially proximate individuals. As Baud et al. note, specific genotypes may have different consequences for an organism’s phenotype depending on the prevalence of that genotype in the social environment (Fig 1, arrow b). Such “social epistasis” may be synergistic, with increasing prevalence of genotypes similar to one’s own amplifying genetic effects. This might be expected in settings where the social environment sets norms for behaviors (e.g., in cases like obesity or educational achievement). Other settings could produce antagonistic interactions, in which increasing prevalence of similar genotypes diminishes or reverses a genetic effect.

These opportunities exist alongside challenges. A primary issue is the extent to which social genotypes are independently determined. Individuals who share traits may be more likely to sort into social units together, a phenomenon called homophily [16,17]. Sorting is also observable at the level of individual genetic loci and polygenic predisposition to certain traits [18,19]. Thus, while SGEs may shape an individual’s phenotype or modify the phenotypic effects of that individual’s genes, reverse causation is also possible; i.e., an individual’s phenotype and/or genotype may shape the genetic composition of their social environment (Fig 1, arrow c). Relatedly, human social relationships are nested within larger social structures [20]. Recent findings in humans and monkeys showing genetic influence on position within society [21] and influence of social position on genomic function [22] suggest accounting for the structural context of social relationship will be important. A final issue is the role of social proximity in conditioning SGEs. In contrast to the mice in Baud et al.’s study, for whom the only social relationship was co-housing, human social relationships span a range of proximities (i.e., spouses as compared to Facebook friends). As human social theory suggests a role for weaker ties in some scenarios [23], research is needed to establish the range of designs in which social genotyping may prove informative.
